# Supplementary material for: Room-temperature coherent manipulation of single-spin qubits in silicon carbide with a high readout contrast
Source: Natl Sci Rev. 2021 Jul 5;9(5):nwab122. doi: 10.1093/nsr/nwab122 (PMC9160373; doi:10.1093/nsr/nwab122)
Supplement: nwab122_Supplemental_File [file nwab122_supplemental_file.pdf]

Supplementary Information: Room temperature coherent manipulation of single-spin qubits in silicon carbide with a high readout contrast

Qiang Li,[1, 2, *] Jun-Feng Wang,[1, 2, *] Fei-Fei Yan,[1, 2] Ji-Yang Zhou,[1, 2] Han-Feng Wang,[1, 2] He Liu,[1, 2] Li-Ping Guo,[3] Xiong Zhou,[3] Adam Gali,[4, 5, †] Zheng-Hao Liu,[1, 2] Zu-Qing Wang,[1, 2] Kai Sun,[1, 2] Guo-Ping Guo,[1, 2] Jian-Shun Tang,[1, 2] Hao Li,[6] Li-Xing You,[6] Jin-Shi Xu,[1, 2, ‡] Chuan-Feng Li,[1, 2, §] and Guang-Can Guo[1, 2]

[1] *CAS Key Laboratory of Quantum Information, University of Science and Technology of China, Hefei, Anhui 230026, People's Republic of China*

[2] *CAS centre for Excellence in Quantum Information and Quantum Physics, University of Science and Technology of China, Hefei, Anhui 230026, People's Republic of China.*

[3] *Key Laboratory of Artificial Micro- and Nano-structures of Ministry of Education and School of Physics and Technology, Wuhan University, Wuhan, Hubei 430072, People's Republic of China.*

[4] *Department of Atomic Physics, Budapest University of Technology and Economics, Budafoki út. 8, H-1111, Hungary*

[5] *Wigner Research centre for Physics, PO. Box 49, H-1525, Hungary*

[6] *State Key Laboratory of Functional Materials for Informatics, Shanghai Institute of Microsystem and Information Technology, Chinese Academy of Sciences(CAS), Shanghai 200050, People's Republic of China*

(Dated: June 23, 2021)

1. PL SPECTRA OF ENSEMBLE AND SINGLE COLOR CENTERS

The material used in our work is a 12.5-μm-thick epitaxial layer of single-crystal 4H-SiC with nitrogen doping density of 5×10^{15} cm^{-3} grown on a 4° off-axis 4H-SiC substrate (Xiamen Powerway Advanced Material Co., Ltd) [1]. We use 30 keV carbon ion implantation to produce divacancies ($V_{Si}V_C$) and divacancy-related defects in 4H-SiC. For the ensemble samples, the implantation dose is 1×10^{13} cm^{-2}, after which we anneal the samples to increase the conversion efficiency.

For the experiments at a low temperature (LT) of 8 K, the SiC samples are mounted on a cooling stage in the Montana Instruments cryostat, and a two-axis Galvo scanning system with silver-coated mirrors (Thorlabs, GVS012) is used to scan the exciting laser. The fluorescence signals filtered by a 1000 nm longpass filter (Thorlabs, FELH1000) is coupled to a single-mode fiber and then guided to a superconducting nanowire single photon detector (SNSPD, Scontel & Photon Technology) to detect the photoluminescence (PL) intensity, or coupled to a multiple mode fibre and then guided to a grating spectrometer (Horiba, iHR 550) with an InGaAs array detector (SMY1-IGA50-512) to measure the PL spectra.

Fig. S1 shows the LT PL spectra of the implanted 4H-SiC samples after annealing with 900 °C for 30 minutes. Because of two inequivalent lattice positions for Carbon and Silicon atoms in 4H-SiC crystals, namely the hexagonal (h) and quasi-cubic (k), there are four kinds of inequivalent identified neutral $V_{Si}V_C$ defects, which are denoted as hh (PL1), kk (PL2), hk (PL3) and kh (PL4). The four apparent peaks (1131.2 nm, 1130.0 nm, 1107.4 nm, 1078.3 nm) in the LT PL spectra of the implanted ensemble samples correspond to the four types of divacancies, which are consistent with previous reports [2, 3]. It is noticed that an unknown peak of 1102.9 nm exists near that of the PL3 defect. There are also three types of divacancy-related defects, which are denoted as PL5, PL6, and PL7. The inset is an enlarged figure of the part shown in the black box, in which the LT PL spectra of PL6 defects are clearly observed. Nevertheless, the LT spectra of PL5 defects are weak due to the low density at these preparing conditions (The PL spectra of PL7 defects have not been identified [3]). Further detailed researches need to deterministically confirm the structures of PL5-PL7 defects and improve their generation efficiency.

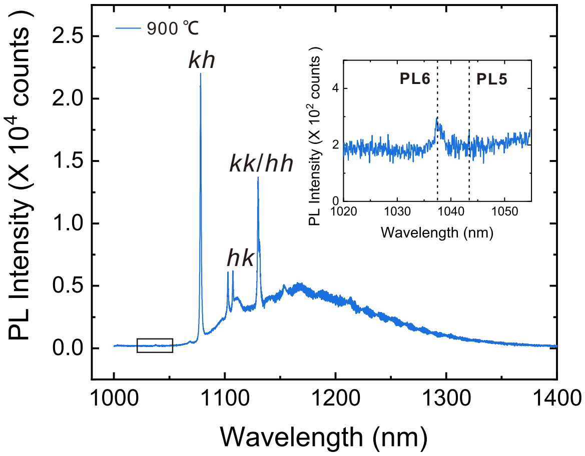

Figure S1. **PL spectra of the ensemble samples at a low temperature of 8 K.** The PL spectra of the implanted samples after annealing with 900 °C for 30 minutes. The inset is an enlarged figure of the part shown in the black box.

The types of divacancies and divacancy-related defects (both denoted as $V_{Si}V_C$) can be identified by optically detected magnetic resonance (ODMR) spectra at room temperature or PL spectra at a low temperature of 8 K.

As previously reported [2, 3], the ODMR spectra of PL1 and PL3 defects in 4H-SiC have been measured, while the ODMR signals of PL2 and PL4 were not observed at room temperature. A grating spectrometer (Princeton, IsoPlane 320) with a low dark count (no more than 8 counts per second) InGaAs camera (NIRvana:640LN) is used to detect the PL spectra of single color centers at 8 K. Fig. S2A and Fig. S2B demonstrate the normalized LT PL spectra of single PL2 and PL4 defects, respectively, while Fig. S2C and Fig. S2D show the LT PL spectra of single PL5 and PL6 divacancy-related defects. The widths of zero-phonon lines of those single defects ranging from 0.42 nm to 0.51 nm, which are limited by the resolution of the grating spectrometer.

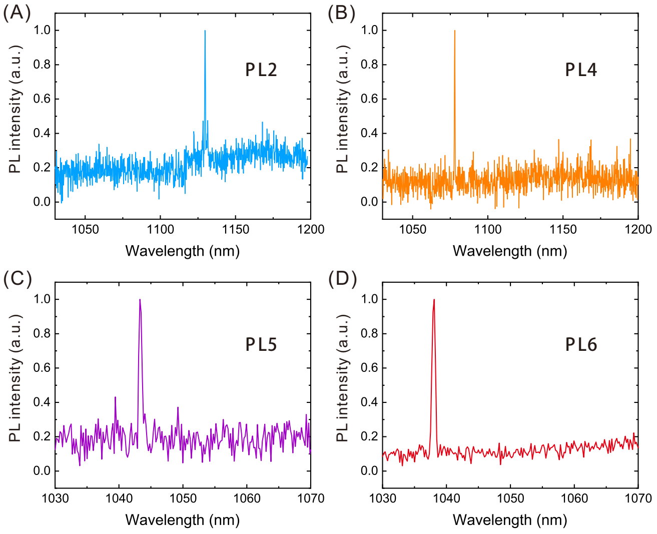

Figure S2. **PL spectra of single color centers at low temperature of 8 K. (A)** and **(B)** demonstrate the PL spectra of single *kk* (PL2, blue) and *kh* (PL4, orange) divacancy defects in the implanted 4H-SiC, respectively. **(C)** and **(D)** demonstrate the PL spectra of single PL5 (purple) and PL6 (red) defects in the implanted 4H-SiC samples.

2. OPTICAL AND SPIN PROPERTIES OF SINGLE PL6 DEFECTS.

We use a Hanbury-Brown and Twiss (HBT) interference device to measure the second-order intensity correlation function $g^{(2)}(t)$ of emitters studied in this work. In the experimental setup, the emitted fluorescence photons are split by a fiber beam splitter into two paths and then detected by a two-chancel superconducting nanowire single photon detectors (SNSPD). A time-to-digital converter (TDC) is used to measure the intensity correlation of the two chancels at different delay time t. The raw experimental result of $g^{(2)}(t)$ of the single PL6 defect shown in Fig. 1A in the main text with an exciting laser power of 0.2 mW is shown in Fig. S3A. The raw value of $g^{(2)}_{raw}(0)$ is measured to be 0.101. In order to eliminate the influence of background fluorescence, the raw data of $g^{(2)}_{raw}(t)$ is corrected with the function:

$$g^{(2)}(t) = [g^{(2)}_{raw}(t) - (1-\varepsilon)]/\varepsilon^2, \qquad \text{(S1)}$$

where $\varepsilon = c/(c+b)$; c and b are the signal and background count rate, respectively [4–6]. The signal-to-noise ratio c/b is measured to be 33. The result with background-correction is shown in Fig. S3B, which is fitted by the function:

$$g^{(2)}(t) = 1 - (1+a)e^{-|t|/\tau_1} + de^{-|t|/\tau_2}, \qquad \text{(S2)}$$

where a, d, τ_1 and τ_2 are the fitting parameters [4–6]. From the fitting, the value of $g^{(2)}(0)$ is deduced to be 0.046, indicating an excellent single photon emitter with relatively high confidence.

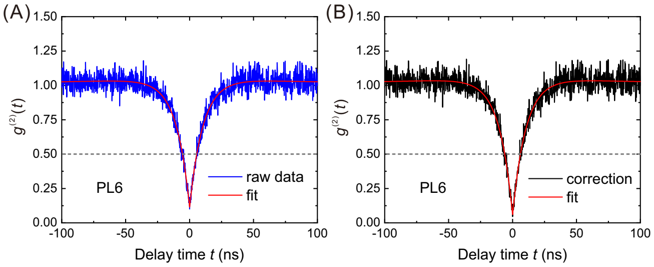

Figure S3. **Second-order intensity correlation function $g^{(2)}(t)$ measurement of a single PL6 defect.** The exciting laser power is set to be 0.2 mW. **(A)** The raw experimental data without correction. **(B)** The result with background-correction.

We also survey a dozen single PL6 defects at room temperature. Fig. S4A shows the distribution of saturated PL intensity of randomly selected single PL6 defects. The saturated PL intensity (background-corrected) ranges from 138.9 kcps to 172.4 kcps with an average value of 155.9 kcps. Fig. S4B shows the

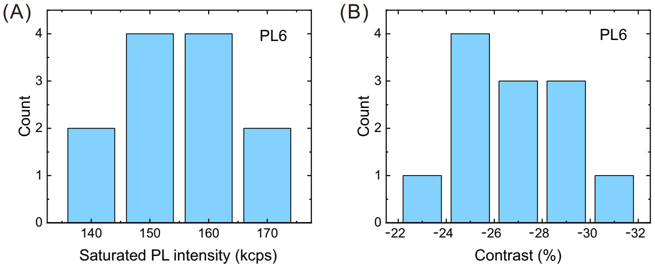

Figure S4. **The saturated PL intensity and Rabi contrast statistics of single PL6 defects at room temperature. (A)** Histogram showing the saturated PL intensity distribution of single PL6 defects. **(B)** Histogram showing the Rabi contrast distribution of single PL6 defects measured with a 0.05-mW laser in a magnetic field of 26.4 G (parallel to the c-axis).

distribution of Rabi oscillation contrast of those single PL6 defect spins excited by a 0.05-mW laser (the readout laser pulse duration is 0.25 µs) at a magnetic field of 26.4 G (parallel to the *c*-axis). The Rabi oscillation contrast ranges from -23.0% to -31.6% with an average value of -26.4%.

3. SPIN-RESOLVED EXCITED STATE LIFETIME FOR SINGLE PL6 AND PL1 DIVACANCY AT ROOM TEMPERATURE.

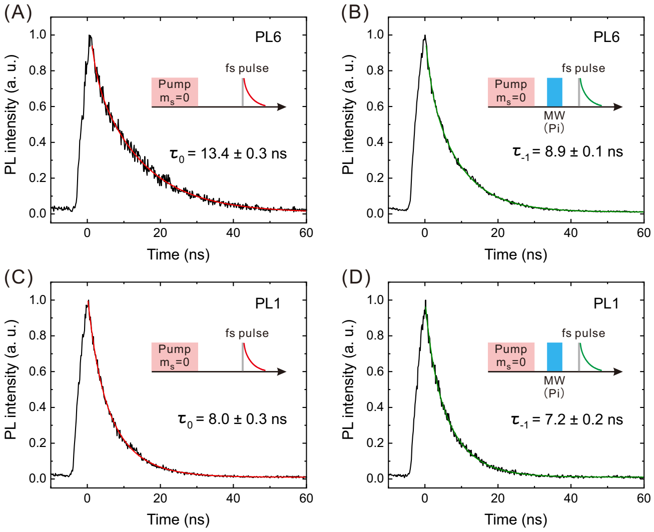

Figure S5. **Spin-resolved excited state lifetime measurements for single PL6 and PL1 defects at room temperature.** **(A)** and **(B)** demonstrate the excited state lifetime of $m_s = 0$ and $m_s = -1$ for a single PL6 defect, respectively. **(C)** and **(D)** show the excited state lifetime of $m_s = 0$ and $m_s = -1$ for a single PL1 defect, respectively.

We measured the spin-resolved ($m_s = 0$ and $m_s = \pm1$) excited state lifetime at room temperature for the single PL6 defect described in the main text. The 920 nm CW laser with a duration of 5 µs is used to polarize the spin state to $m_s = 0$. After waiting for 1 µs for the singlets to depopulate, the spin would be initialized to the ground sate $m_s = 0$. The single PL6 defect is then further excited by a 150 fs laser pulse, and the fluorescence decay is recorded [7]. The measurement strategy and experimental results are shown in Fig. S5A. The fluorescence decay curve is fitted by a double exponential decay function, in which one of the time parameters is the excited state ($m_s = 0$) lifetime, and the other fast decay may originate from the system response or background fluorescence decay [8]. From the fitting, the excited state ($m_s = 0$) lifetime of the single PL6 defect is deduced to 13.4 ± 0.3 ns. As shown in Fig. S5B, after initialized into $m_s = 0$, the spin state is then converted into $m_s = -1$ by a microwave π-pulse. The fluorescence decay is further recorded, from which the excited state lifetime of $m_s = -1$ is deduced to 8.9 ± 0.1 ns. The spin-resolved excited state lifetime for the single PL1 defect described in the main text is measured for comparison, which is shown in Fig. S5C and S5D. The excited state lifetime of $m_s = 0$ and $m_s = -1$ are deduced to 8.0 ± 0.3 ns and 7.2 ± 0.2 ns, respectively.

4. OPTIMIZING THE CW-ODMR MEASUREMENT.

For the application of magnetometer, the continuous-wave (CW)-ODMR is an immediate and straightforward method to sense dc magnetic field through estimating the value of Zeeman splitting. We systematically study the influences of exciting laser power and microwave (MW) power on the CW-ODMR contrast and linewidth of a single PL6 defect spin at room temperature, which is similar to the previously reported work for NV centers in diamond [9]. The ON-OFF measurement scheme is the same as our previous work described in [10]. A 50-µm copper wire above the sample is used to radiate microwave. The step size of the microwave scan is set to be 1 MHz, and the scan process is implemented 10 times, after which the results are averaged. For each point, the microwave is gated on and off with 1 ms duration and repeated 1000 times. Each result is fitted by a Lorentz function. Fig. S6A shows the CW-ODMR contrast as a function of Rabi frequency (Rabi frequencies increase linearly with the square root of the microwave power on the copper wire, as shown in Fig. S6E) at different exciting laser powers measured at the magnetic field of 23.7 G (parallel to the *c*-axis). The results demonstrate that the ODMR contrast significantly increases with the increase of Rabi frequency (microwave power) at the same exciting laser power. Fig. S6B shows the CW-ODMR linewidth of the single PL6 defect spin as a function of Rabi frequency at different exciting laser powers. The experimental results demonstrate that the linewidth decreases as Rabi frequency (microwave power) decreases at the same exciting laser power. Fig. S6C shows the CW-ODMR contrast of the single PL6 defect spin as a function of laser power with different Rabi frequencies (microwave powers). The results demonstrate that the CW-ODMR contrast increases with the decrease of laser power. Fig. S6D shows the CW-ODMR linewidth of the single PL6 defect spin as a function of laser power with different Rabi frequencies (microwave powers). The results demonstrate that the linewidth decreases with the decrease of laser power. Fig. S6E shows the Rabi frequencies of the single PL6 defect spin as a function of the square root of the microwave power on the copper wire. The Rabi frequencies increase linearly with the square

root of the microwave power (the solid red line is a linear fit). Fig. S6F shows one of the optimized examples, that is the CW-ODMR spectra excited by a 0.05 mW laser and 4.27 W microwave (corresponding to 4.26 MHz Rabi frequency) at a magnetic field of 23.7 G. The experimental data (blue dots) is fitted by a two-peak Lorentzian function centering at 1.2850 GHz and 1.4179 GHz, respectively (solid orange line), from which the ODMR contrast and linewidth are deduced to be $-18.5\% \pm 0.3\%$ and 12.8 ± 0.4 MHz, respectively.

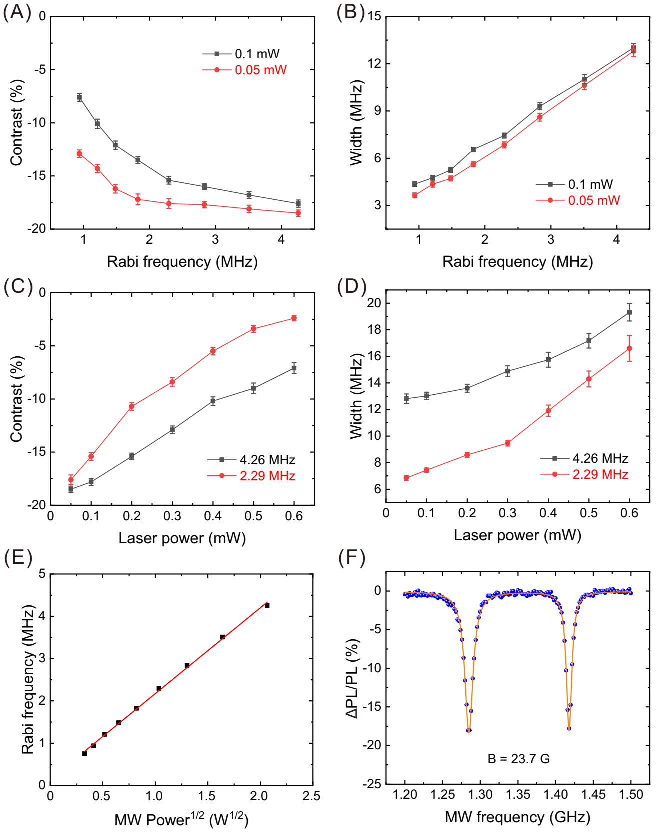

Figure S6. **CW-ODMR contrast and linewidth depending on the power of exciting laser and microwave at room temperature. (A)** The ODMR contrast of a single PL6 defect spin as a function of Rabi frequency exciting with 0.05- (red) and 0.1-mW (black), 920-nm CW laser, respectively. **(B)** The ODMR linewidth of the single PL6 defect spin as a function of Rabi frequency excited with 0.05- (red) and 0.1-mW (black), 920-nm CW laser, respectively. **(C)** The ODMR contrast of the single PL6 defect spin as a function of laser power with 2.29 MHz (red) and 4.26 MHz (black) Rabi frequency, respectively. **(D)** The ODMR linewidth of the single PL6 defect spin as a function of laser power with 2.29 MHz (red) and 4.26 MHz (black) Rabi frequency, respectively. **(E)** Rabi frequency as a function of the square root of the microwave power on the copper wire. The solid red line is a linear fit. **(F)** CW-ODMR spectra of a single PL6 defect excited by a 0.05-mW laser and 4.27 W microwave (corresponding to 4.26 MHz Rabi frequency) at a magnetic field of 23.7 G (parallel to the c-axis).

5. DYNAMICAL DECOUPLING OF SINGLE PL6 DEFECT SPINS.

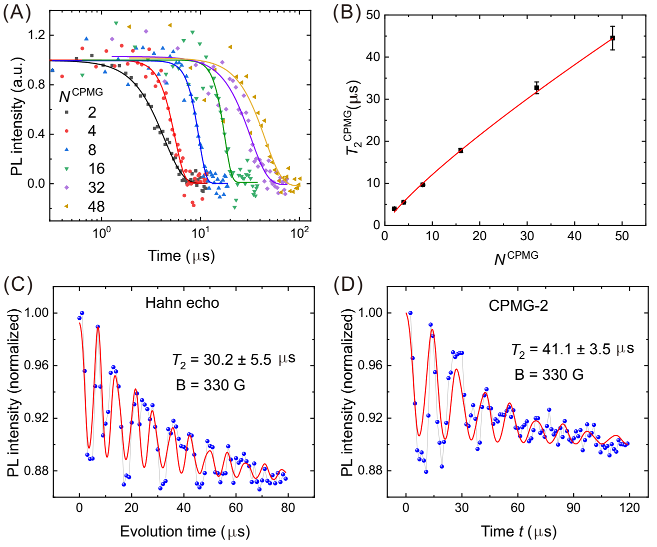

Figure S7. **Dynamical decoupling of single PL6 defect spins. (A)** Short-time coherence decay along the refocusing rotation axis with CPMG-N sequences measured in a magnetic field of 330 G. **(B)** Short-time coherence decay characteristic time T_2^{CPMG} as a function of the number of π-pulses N^{CPMG}. **(C)** Hahn echo measured in a magnetic field of 330 G, from which the coherence time T_2 is deduced to be 30.2 ± 5.5 μs. **(D)** The total time evolution with the application of CPMG-2 sequence measured in a magnetic field of 330 G. From the fitting, the coherence time T_2 is deduced to be 41.1 ± 3.5 μs.

The coherence time is essential to proposals for using single electronic spins in quantum information processing or sensing. We use Carr-Purcell-Meiboom-Gill (CPMG) decoupling sequences [11] to prolong the spin coherence time T_2 of single PL6 defects. The N-π pulses CPMG sequences are used in this work. The CPMG sequences with a different number of π-pulses are applied to a single PL6 defect in a magnetic field of 330 G. The short-time coherence decay of the single electronic spin for different N^{CPMG} are demonstrated in Fig. S7A. The dots are experimental results of PL intensity as a function of evolution time. The solid lines are corresponding fits to $Ae^{(-t/T_2^{\mathrm{CPMG}})^n}$ with free parameters A and n. As the number N^{CPMG} increases, the coherence time is extended. The coherence characteristic time T_2^{CPMG} with different N^{CPMG} are summarized in Fig. S7B. The red solid line is fit to the function of $T_2^{\mathrm{CPMG}}(N^{\mathrm{CPMG}}) = B(N^{\mathrm{CPMG}})^{\alpha}$, in which the fitting parameter $\alpha = 0.78 \pm 0.05$ and B is the free parameter. The short-time coherence decay time T_2^{CPMG} is extended by 10.3 times (from 4.3 ± 0.1 μs to 44.5 ± 2.5 μs). For comparison, the Hahn echo sequence is applied under the same condition, and the results are demonstrated in

Fig. S7C. The blue dots are experimental results, and the solid red line is the corresponding fitting curve, from which the Hahn echo coherence time T_2 is deduced to be 30.2 ± 5.5 μs. The total time evolution of CPMG-2 measurement is shown in Fig. S7D, from which the coherence time T_2 measured with CPMG-2 sequences is deduced to be 41.2 ± 3.5 μs. The coherence time T_2 is extended by 1.36 times.

6. THE PULSE SEQUENCE TO MEASURE THE LONGITUDINAL COHERENCE TIME T_1.

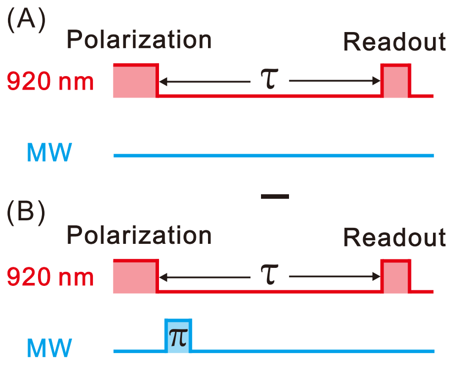

Figure S8. T_1 **measurement sequence.** Optical and microwave pulse sequences used for T_1 measurement. Difference counts between these two process **(A)** and **(B)** are used to derive T_1.

The optical and microwave pulse sequences shown in Fig. S8 are used to measure the longitudinal coherence time T_1 of a single PL6 defect spin at room temperature, which is similar to the previous work [12]. In each sequence, the initial state is polarized into the state of $m_s = 0$ by applying a 920 nm laser pulse for 20 μs. There is a microwave π pulse with a typical duration of 66 ns after the initialization in Fig. S8B, in which the initial state is then rotated to $m_s = -1$. After a decay time τ, a laser pulse with a length of 0.55 μs is used to detect the PL intensity, which depends on the residual spin polarization. The different PL intensity between these two processes as a function of τ is used to derive the longitudinal coherence time T_1.

7. OPTICAL AND SPIN PROPERTIES OF SINGLE PL5 DEFECTS.

We characterize the optical properties of single PL5 defects. Fig. S9A shows a representative raw data of $g^{(2)}_{raw}(t)$ for a single PL5 defect excited with a 1-mW CW laser. From the raw data, the value of $g^2_{raw}(0)$ is deduced to be 0.145, indicating a single emitter. In order to eliminate the influence of background fluorescence, $g^2_{raw}(t)$ is corrected with the function of Eq. (S1). The results are shown in Fig. S9B. The background-corrected $g^2(t)$ is fitted by the function of Eq. (S2). From the fitting, the value of $g^{(2)}(0)$ is deduced to be 0.0012, indicating an excellent single-photon emitter with fairly high confidence. We then measure its saturation behavior excited with a 920-nm CW laser, as shown in Fig. S9C. The blue rhombuses are the background-corrected experimental data and fitted by the function:

$$I(P) = I_s \cdot P/(P + P_s), \tag{S3}$$

where $I(P)$ and P are the corresponding count rate and exciting power, with I_s and P_s being the saturated count rate and saturated exciting power. From the fitting, the saturated exciting power P_s and the saturated count rate I_s are deduced to be 0.67 ± 0.01 mW and 63.8 ± 0.3 kcps, respectively.

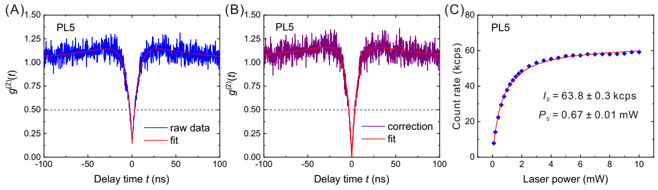

Figure S9. **Optical properties of a single PL5 defect at room temperature.** The second-order intensity correlation function ($g^{(2)}(t)$) of the single PL5 defect without **(A)** or with **(B)** background-correction for exciting laser power of 1 mW. The blue and purple lines are the raw data and corresponding results with background correction, respectively. The solid red lines are corresponding fittings to the function of $g^{(2)}(t)$. **(C)** Saturation behavior. The blue rhombuses are the background-corrected experimental data, and the solid red line is the fitting with a function of Eq. (S3). The saturated exciting power P_S and the saturated count rate are deduced to be 0.67 ± 0.01 mW and 63.8 ± 0.3 kcps, respectively.

We further survey the saturated PL intensity and Rabi oscillation contrast of various single PL5 defects at room temperature. Fig. S10A shows the distribution of saturated PL intensity of randomly selected single PL5 defects. The saturated PL intensity (background-corrected) ranges from 52.9 kcps to 84.1 kcps with an average value of 69.5 kcps. Fig. S10B shows the distribution of Rabi oscillation contrast of these single PL5 defect spins excited by a 0.05-mW laser (the readout laser pulse duration is 0.25 μs) without a magnetic field. The Rabi oscillation contrast ranges from -23.6% to -28.5% with an average value of -26.0%.

We also characterize the longitudinal coherence time T_1, as shown in Fig. S11. From the fitting of single-exponential decay, the T_1 time is deduced to be 158.6 ± 17.5 μs.

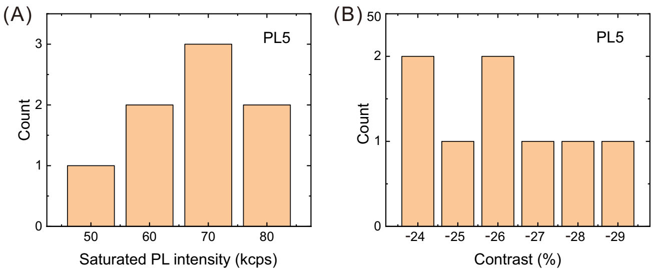

Figure S10. **Saturated PL intensity and Rabi oscillation contrast statistics of single PL5 defects at room temperature. (A)** Histogram showing the saturated PL intensity distribution of single PL5 defects. **(B)** Histogram showing the Rabi oscillation contrast distribution of single PL5 defects excited by a 0.05-mW laser without a magnetic field.

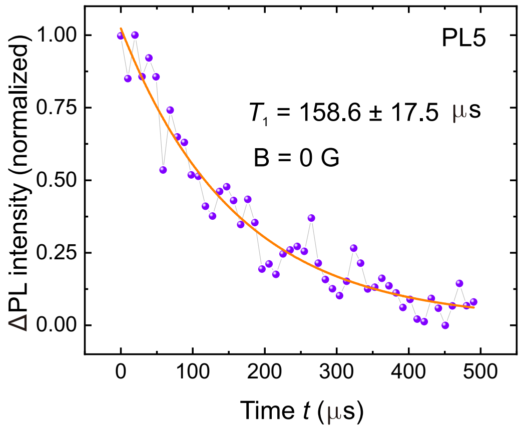

Figure S11. **Longitudinal coherence time T_1 of a single PL5 defect at room temperature.** Longitudinal coherence time is measured without a magnetic field. From the fitting of single-exponential decay, the T_1 time is deduced to be 158.6 ± 17.5 μs.

8. OPTICAL AND SPIN PROPERTIES OF SINGLE PL1 AND PL7 DEFECTS.

We characterize the optical properties of single PL1 defects. Fig. S12A shows the raw data of $g^{(2)}_{raw}(t)$ for a representative single PL1 defect excited with a 1-mW CW laser. From the raw data, the value of $g^{2}_{raw}(0)$ is deduced to be 0.27, indicating a single emitter. In order to eliminate the influence of background fluorescence, $g^{2}_{raw}(t)$ is corrected with the function of Eq. (S1). The result is shown in Fig. S12B. The background-corrected $g^2(t)$ is fitted by the function of Eq. (S2). From the fitting, the value of $g^{(2)}(0)$ is deduced to be 0.036, indicating an excellent single-photon emitter. Then, we measure its saturation behavior excited with a 920-nm CW laser, as shown in Fig. S12C. The black squares are the background-corrected experimental data and fitted by the function of Eq. (S3), from which the saturated exciting power P_s and the saturated count rate I_s are deduced to be 2.2 ± 0.1 mW and 72.1 ± 1.1 kcps, respectively.

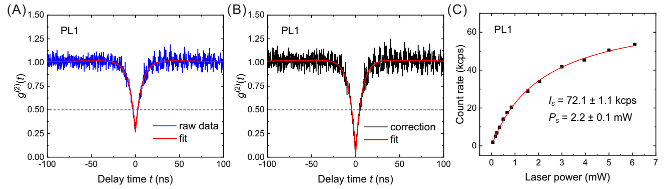

Figure S12. **Optical properties of a single PL1 defect at room temperature.** The second-order intensity correlation function ($g^{(2)}(t)$) of the single PL1 defect without **(A)** or with **(B)** background-correction for exciting laser power of 1 mW. The blue and black lines are the raw data and corresponding results with background-correction, respectively. The solid red lines are corresponding fitting to the function of $g^{(2)}(t)$. **(C)** Saturation behavior. The black squares are the background-corrected experimental data, and the solid red line is the fitting with a function of Eq. (S3). The saturated exciting power P_S and the saturated count rate are deduced to be 2.2 ± 0.1 mW and 72.1 ± 1.1 kcps, respectively.

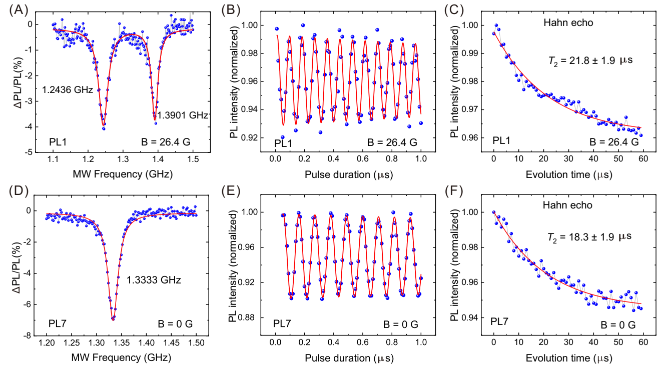

Figure S13. **Spin properties of single PL1 and PL7 defects at room temperature. (A-C)** Spin properties of a single PL1 defect measured in a magnetic field of 26.4 G. **(A)** represents the CW-ODMR spectra of a single PL1 defect spin. The blue dots are experimental raw data, and the red lines represent corresponding Lorentzian-shaped multi-peak fittings. **(B)** represents Rabi oscillations of the single PL1 defect spin. **(C)** represents Hahn echo coherence time T_2 of the single PL1 defect spin. From the fitting, the homogeneous spin coherence time T_2 is deduced to be 21.8 ± 1.9 μs. **(D-F)** Spin properties of a single PL7 defect measured without a magnetic field. **(D)** represents the CW-ODMR spectra of a single PL7 defect spin. **(E)** represents Rabi oscillations of the single PL7 defect spin. **(F)** represents Hahn echo coherence time T_2 of the single PL7 defect spin. From the fitting, the homogeneous spin coherence time T_2 is deduced to be 18.3 ± 1.9 μs.

We investigate the spin properties of single PL1 and PL7 defects at room temperature. Fig. S13A-C show the spin properties of a representative single PL1 defect at room temperature measured in a magnetic field of

26.4 G, which is arranged to be parallel to the c-axis. Fig. S13A shows the ODMR spectra of the single PL1 divacancy in the magnetic field of 26.4 G excited with 50-μW laser pumping. The contrast of the CW-ODMR spectra is deduced to be about −4%. Fig. S13B demonstrates the Rabi oscillation in the magnetic field of 26.4 G, where the contrast is deduced to be about -6.6%. Fig. S13C shows the Hahn echo coherence time T_2 of the single PL1 defect in the magnetic field of 26.4 G at room temperature. The blue dots are experimental results of normalized PL intensity as a function of total evolution time. The solid red line is the fitting result of a single exponential decay function. From the fitting, the homogeneous spin coherence time T_2 is deduced to be 21.8 ± 1.9 μs. Fig. S13D-F show the spin properties of a single PL7 defect at room temperature measured without a magnetic field. Fig. S13D and Fig. S13E exhibit the zero-field CW-ODMR spectra and Rabi oscillation of a single PL7 defect with 0.2-mW laser pumping, respectively. The oscillation frequency is deduced to be 1.3333 GHz. The contrasts of CW-ODMR and Rabi oscillation are about −7% and -10%, respectively. Fig. S13F shows the Hahn echo experimental results of the single PL7 defect spin measured without a magnetic field at room temperature, from which the Hahn echo coherence time T_2 is measured to be 18.3 ± 1.9 μs.

9. STATISTICS OF DIVACANCY IN THE IMPLANTED 4H-SIC SAMPLE

According to previously reported works, single PL3 defects were not found in the 4H-SiC sample via electron radiation [8, 13, 14]. We used 30-keV carbon ion implantations and annealing to generate single divacancy defects in the 4H-SiC sample in this work. Although other types of divacancies were observed, we also did not find single PL3 centers in the samples implanted by C ions at room temperature. The types of single divacancy in 4H-SiC generated by electron or C ion implantations are summarized in Table S1. The underlying cause, why the single PL3 defects in 4H-SiC have not been or were difficult to find, needs further study.

For comparison, we also summarized some essential information of PL1-PL7 divacancy in Table S1, including the atom structure, orientation, spin, and charge state of the bright state, ZPL at cryogenic temperature, character peaks of ODMR spectra at room temperature, etc.

According to Ref. [17], the authors suggested that defects like PL5, PL6, and PL7 exist near the stacking faults, which are near the surface of 4H-SiC. To estimate the distribution of the PL5, PL6, and PL7 single defects, we randomly selected a 10 × 10 array of implanted sites and distinguished a single PL5-PL7 defect through ODMR spectra and second-order intensity correlation function ($g^{(2)}(t)$) measurements. In the 100 implanted sites, the number of the single PL5, PL6, and PL7 defects is 7, 1, and 6, respectively, from which the PL5, PL6, and PL7 single defects account for 7%, 1%, and 6% of the implanted sites, respectively. The results are shown in Fig. S14.

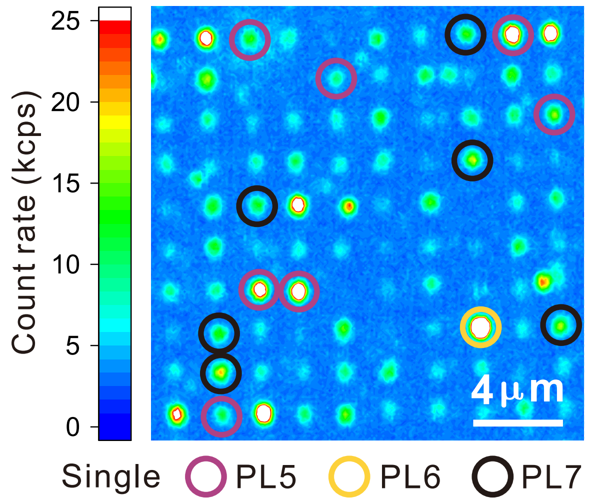

Figure S14. Distribution of single PL5, PL6, and PL7 defects in a 10 × 10 array of implanted sites. The purple, orange, and black cycles represent the single PL5, PL6, and PL7 defects, respectively. The number of the single PL5, PL6, and PL7 defects found in the area is 7, 1, and 6, respectively.

10. THEORY ON THE OPTICAL DETECTED MAGNETIC RESONANCE READOUT OF DIVACANCY DEFECTS IN 4H-SIC

Decay pathways

The decay paths connect the initial and final states that are labeled by numbers in Fig. 5A of the main text. The radiative transition between the different spin levels is very weak, e.g., r_{41} and r_{32} rates are small, because it is caused only by tiny spin-spin interaction within the corresponding ^3E manifold (see Ref. 18 and references therein). Therefore, these rates can be neglected. Very likely, the direct non-radiative decay between the same spin levels of the triplets (green dotted arrows) is weak compared to the radiative decay (straight red arrows) because of ∼ 1.1 eV gap between the triplet states of the divacancy defects. They both contribute to the direct decay from the ^3E excited state to the $^3A_2$ ground state.

The calculated energy gap between $^1\widetilde{E}$ and ^3E is about 0.8 eV for divacancy defects [19]. The estimated accuracy of the applied method is about 0.1 eV. Taking this into account, one can safely conclude that the energy spacing between $^1\widetilde{E}$ and ^3E cannot be efficiently bridged by phonons. In other words, the phonon overlap spectral function F between these states is nearly zero at this en-

PL name	Structure	Orientation	Spin	Charge state	ZPL (nm)	ODMR1 *(GHz)	ODMR2 *(GHz)	Electron radiation	Carbon ion implantation
PL1	hh	c-axis	1	0	1132	1.317	-	yes	yes
PL2	kk	c-axis	1	0	1131	-	-	yes	yes
PL3	hk	basal	1	0	1108	-	-	-	-
PL4	kh	basal	1	0	1078	-	-	yes	yes
PL5	k_1h_1	basal	1	0	1043	1.344	1.376	-	yes
PL6	k_2k_2	c-axis	1	0	1038	1.351	-	-	yes
PL7	k_2k_1	basal	1	0	-	1.333	-	-	yes

* Room temperature results in a zero magnetic field

TABLE S1. **Properties of divacancies in 4H-SiC.** The data of structure, orientation, spin state and zero-photon-line (ZPL) for PL1-PL7 defects are obtained from Ref. [3, 13, 17]. The data of charge state are obtained from Ref. [15–17]. The data of the resonant position of the ODMR spectra for PL1-PL7 in a zero magnetic field at room temperature are from this work. The types of single divacancy in 4H-SiC generated by the electron radiation or Carbon ion implantation are obtained from Ref. [8, 13] or this work, respectively.

ergy (see the definition of F in Ref. 18 and references therein). F is non-zero between ^3E and $^1\widetilde{A}_1$ states as well as between $^1\widetilde{E}$ and $^3A_2$ states. The spin-flipping processes, responsible for optical spin-polarization and ODMR readout contrast, should be studied between these states.

Understanding the nature of the singlet states lying in between the triplet states is key for determining the strength of the optical spin-polarization and ODMR readout contrast. Here we focus on the analysis of the ODMR readout contrast, but the optical spin-polarization is also briefly discussed in the context. In the lower branch of the spin-polarization loop, selection rules imply a non-trivial connection between states 5 and 2. This can occur if one considers the correlation between the high energy $^1E'$ and the low energy ^1E states separated by an energy gap of Δ (see the box at the right side of Fig. 5A of the main text) which can then connect states 5 and 2 by the perpendicular component of the spin-orbit coupling. However, this goes opposite to the observations on NV centre in diamond that found spin-polarization to the $|0\rangle$ state (state 1), which requires $r_{51} > r_{52}$. A recent theory explained this phenomenon by the strong electron-phonon coupling between the ^1E and $^1A_1$ states separated by Λ, which is beyond the Born-Oppenheimer approximation [20]. The electron-phonon coupling between ^1E and $^1A_1$ states can be treated within the so-called pseudo Jahn-Teller theorem, and the electron-phonon coupling results in dynamic Jahn-Teller effect for the $^1E'$ state which is mixed into ^1E by electron correlation (see Ref. 18 and references therein). As a consequence, $^1A_1$ mixes into ^1E which makes it possible to connect the lower singlet state to the $|0\rangle$ state by the parallel component of the spin-orbit coupling (λ_z). Because of the pseudo Jahn-Teller effect, the singlet states between the triplet states are vibronic states that are labelled by tilde (see the box in the middle of Fig. 5A of the main text).

Divacancy in 3C-SiC revisited.

Single divacancy measurements have been carried out in 3C-SiC in details [8] which is supposed to be the closest system to NV centre in diamond, in terms of electronic structure and crystalline environment. The observed ODMR contrast upon off-resonant excitation was found at -7.5% at low temperatures. Time-dependent PL decay studies showed biexponential components where the faster rate was associated with the decay from $ms = \pm 1$ "dark" state, whereas the slower rate was associated with the decay from $ms = 0$ "bright" state. They applied a five-level model (states 5 and 6 were considered as a single effective state in Fig. 5A), and they carried out a fitting procedure on the observed ODMR contrasts and power-dependent optical spin-polarization and PL intensities. Finally, they conclude that $r_{36} = 11$ MHz is in the same order of magnitude as $r_{46} = 21$ MHz for divacancy defect in 3C-SiC which can explain the -7.5% ODMR contrast and 96% optical spin-polarization of the electron spin. They assumed that r_{52} is negligible in the fitting procedure.

After this report, results from the novel theory on the optical spin-polarization loop [20] and the accurate electronic structure calculations of divacancy SiC [19] may question the interpretation of the observed biexponential decay. The only link between $\left|^3E\right\rangle$ $ms = 0$ (state 3) and the singlet states is the λ_z spin-orbit component towards $\left|^1E'\right\rangle$. Therefore, first order decay ISC process is only possible towards such lower lying singlet state in which the $\left|^1E'\right\rangle$ component is substantial.

It has been found (see Supplementary Information in Ref. 19) that $\left|^1E'\right\rangle$ only appears in $\left|^1\widetilde{E}\right\rangle$ (state 5) and it is marginal (not detectable) in $\left|^1\widetilde{A}_1\right\rangle$ (state 6). As a consequence, rather r_{52} should be significant via $\lambda_\perp$

interaction whereas r_{36} should be very minor, and definitely cannot be similar order of magnitude with that of r_{46}. Principally, r_{35} ISC may occur but the large energy gap between $\left|{}^1\widetilde{E}\right\rangle$ and $\left|{}^3E\right\rangle$ makes it also tiny and not comparable to r_{46}. The main conclusion is that the optical spin-polarization loop in divacancy defects in SiC should be very similar to that of diamond NV centre.

By assuming these conditions, one can apply Eq. (1) in the main text with the observed $1/r_0 = \tau_0 = 18.7\pm0.3$ ns and $1/r_{\pm1} = \tau_{\pm1} = 15.8 \pm 0.3$ ns optical lifetimes on 3C-SiC single divacancy defect [8]. By taking the average values, one yields $C = -0.155 = -15.5\%$ which is stronger than the observed off-resonant pulsed ODMR readout contrast at -7.5%. Again, it is demonstrated that our theory yields an upper bound limit for the absolute value of the contrast and it implies that there is a room for improvement in the parameters of the readout protocol for strengthening the observed contrast.

The ODMR readout contrasts of divacancy defects at low temperature.

In diamond NV centre, the $F_A(E)$ is sizable up to about $E = 0.4$ eV and then it decays exponentially. As $\Omega \approx 0.4$ eV (see Ref. 18 and references therein), this leads a relatively high r_{46} rate. According to Ref. 19, $\Omega = 0.08\ldots0.15$ eV for the PL1-4 configurations in 4H-SiC which fall into the energy region where the exponential tail in F_A starts as can be seen the exponential tail in the phonon side band with respect to the ZPL position of the PL1-4 PL spectra (e.g., Fig. S1).

We turn to the quantitative differences of the ODMR readout contrasts of divacancy configurations in 4H-SiC in light of the previous considerations. The low-temperature CW-ODMR readout contrasts for the PL1-4 defects were around -18%. However, they reached about twice as high values for PL5 and PL6 defects as observed by off-resonant excitation of ensembles [21]. PL5 and PL6 defects have been recently identified as divacancy configurations in the middle of the stacking fault which can be seen as a small 6H SiC polytype inclusion [17]. As a consequence, a local triangular quantum well is formed around the defect which localizes the electronic states. It is emphasized that the PL5 and PL6 defects are chemically the same compositions as the PL1-4 divacancy defects. Thus, one can argue that the localization of the electrons caused by the quantum well results in a larger separation between the corresponding electronic states for PL5 and PL6. Indeed, the observed ZPL energies for PL5 and PL6 are larger than those for PL1-4 configurations outside the quantum well. Direct *ab initio* calculation of the singlet states is computationally prohibitive because a large model is required to embed the divacancy into a stacking fault. Nevertheless, the trends can be drawn by the arguments of the confined electronic states and larger energy gaps. A smaller contribution of $\left|{}^1A\right\rangle$ and $\left|{}^1E'\right\rangle$ in $\left|{}^1\widetilde{E}\right\rangle$ occurs for PL5 and PL6 defects because the strength of electron-phonon interaction and the correlation of the electronic states scales with the inverse of Λ and Δ, respectively. It cannot be definitely estimated from the quantum confinement argument how the relative energy positions change between the triplet and singlet states such as Ω. Nevertheless, it is likely that the quantum confinement acts stronger on the singlet states than on the triplet states because the singlet states do not have a compensating effect due to exchange interaction between the interacting electrons like the triplet states do. If this gap (Ω) closes, then the absolute value of r_{46} ISC rate will increase because of the larger F_A values which finally results in a larger ODMR readout contrast. For example, estimating from the PL phonon sideband of the PL1 defect, $E \sim 0.05$ eV smaller energy in $F_A(E)$ can result in a $3\times$ factor of $F_A(E)$ in the exponential tail region of the $F_A(E)$ function. This will then increase r_{46} by $3\times$.

Temperature dependence of the ODMR readout contrast for PL5 defect.

The temperature dependence of the lifetime of $\left|{}^1\widetilde{E}\right\rangle$ is now well understood by the vibronic levels and states [20]. The first excited vibronic state, $\left|\widetilde{A}_1\right\rangle$ (state $5'$), which lies at δ energy above the $\left|{}^1\widetilde{E}\right\rangle$ level, has a larger component of $\left|{}^1A_1\right\rangle$ and $\left|{}^1E'\right\rangle$. At elevated temperatures $\left|\widetilde{A}_1\right\rangle$ is getting occupied and opens a faster decay channel towards the ground state manifold (states 1 and 2). This process is important to evaluate the temperature dependence of the optical spin-polarization and the optical pumping rate. The trend is that δ increases going from PL1-4 defects to PL5/6 defects because of the quantum confinement which may suppress the temperature dependence of the lifetime of $\left|{}^1\widetilde{E}\right\rangle$ for PL5 and PL6 defects.

The temperature dependence of the ODMR readout contrast of PL5 defect can be studied by combining results in the literature and the present study. The CW-ODMR readout contrast of ensembles of PL5 defect was measured at elevated temperature where the background PL signal was relatively high, which resulted in a much lower CW-ODMR readout contrast at room temperature (-2.4%) (see Ref. 10) than that in single defect measurement (-18.0%, this work). We note that this phenomenon is common with the diamond NV centre. This is about a factor of 7.5 for PL5 defect in the two studies. We assume that the same factor applies to the CW-ODMR readout contrast as detected in the ensemble measurements to generate the CW-ODMR readout contrasts for a single PL5 defect at elevated temperatures. At low temperatures (~ 20 K), the CW-ODMR readout

contrast was derived from the change in the zero-phonon-line where the signal-to-noise ratio is much improved compared to the detection in the phonon sideband with overlapping PL spectra of other defects [21]. Finally, the derived CW-ODMR readout contrast was at −24% and the observed PL lifetime was at around 13 ns [21]. The observed CW-ODMR contrast is generally lower than the observed pulsed ODMR contrast (e.g., see the Supplementary Information of Ref. 21). In the present study, the CW-ODMR contrast is −18%, whereas the pulsed ODMR contrast is −26% for a single PL5 centre at room temperature (average value). This gives an estimate of about a factor of 1.444 enhancement from CW-ODMR contrast to pulsed ODMR contrast which is in order with the estimate in Ref. 21. Thus, the high-temperature ensemble CW-ODMR contrasts in Ref. 10 should be multiplied by 10.833 to generate the single defect pulsed ODMR contrasts, whereas the low-temperature single defect CW-ODMR readout contrast at −24% should be multiplied by 1.444 to generate the pulsed ODMR readout contrast (about −34.7%), to comply with our particular experimental setup and pulse protocol. By having the low temperature pulsed ODMR readout contrast and optical lifetime in our hand, we can use the simple equation derived for pulsed ODMR readout contrast (Eq. (1) in the main text).

It is assumed that the observed optical lifetime at 13 ns belongs to the bright transition between the $ms = 0$ spin states of the triplets. This sets the value of $\tau_0(T \approx 0\text{K})$ in Eq. (2) of the main text for PL5 defect. The rate r_{46} can be deduced by reproducing the low temperature pulsed ODMR readout contrast which resulted in $r_{46} = 41$ MHz for PL5 defect (see Eq. (1) of the main text). The corresponding lifetime is $\tau_{\pm 1} = 8.5$ ns. Having these parameters in our hand, we fit the W and s parameters in the Mott-Seitz model by using Eq. (2) of the main text (see also Fig. 5B) to reproduce the observed ODMR readout contrasts as a function of temperature as explained above. Finally, $W = 0.076 \pm 0.003$ eV and $s = 2.00 \pm 0.16$ are obtained for PL5 defect in the fit (see Fig. S15).

The results imply that the ODMR readout contrast of PL5 starts to shrink already at $\sim$ 125 K because of the relatively small $W \approx 0.08$ eV (c.f., diamond NV centre with $W \approx 0.5$ eV). This W value is consistent with the results of previous *ab initio* calculations [19], where $W = 0.1$ eV obtained for PL1 defect. These results are consistent with our models and assumptions. By assuming similar adiabatic potential energy surface curvatures for the ^3E and $^1A_1$ states in the PL1 and PL5 defects, smaller Ω for PL5 defect due to quantum confinement implies smaller W too for PL5 defect than those for PL1 defect.

We apply the same procedure to study the temperature dependence of the ODMR readout contrast for PL1 defect. In this case, only the low-temperature data from Ref. 21 (derived contrast is at −14.5%) and our room temperature data (−6.6%). We use the observed optical lifetime at 15 ns belongs to the bright transition between the $ms = 0$ spin states of the triplets [21]. This sets the value of $\tau_0(T \approx 0\text{K})$ in Eq. (2) of the main text for PL1 defect. We derive $\tau_{\pm 1} = 12.83$ ns from Eq. (1). We fix $W = 0.1$ eV as obtained from first principles calculation [19], and we only fit s in Eq. (2) to plot the temperature dependence as shown and compared to that of PL5 defect in Fig. S16. We obtain $s = 4.29$ in this procedure. The temperature dependence on the readout contrast of PL1 defect starts at higher temperatures than that of PL5 defect but then it changes more radically upon elevated temperatures than that of PL5 defect.

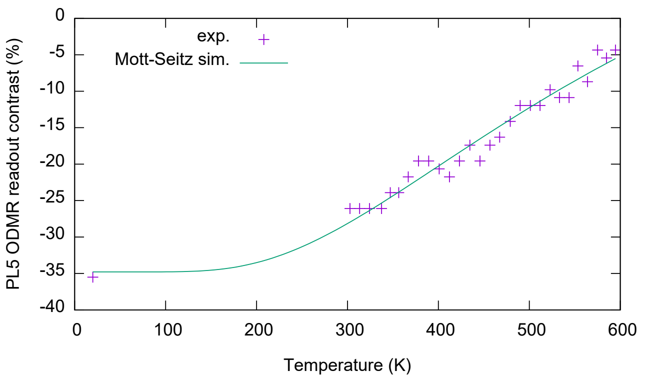

Figure S15. **Temperature dependence of the ODMR readout contrast for PL5 defect.** The experimental data (exp.) are taken from the combination of three studies as explained in the text. The simulation curve is produced by fitting two parameters into Eq. (2) of the main text to produce the ODMR readout contrast as defined in Eq. (1) of the main text.

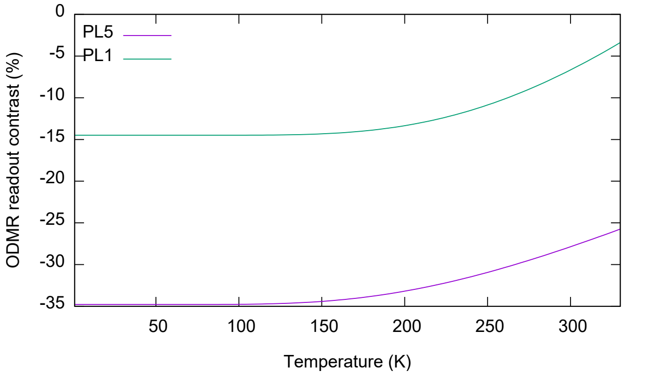

Figure S16. **Temperature dependence of the ODMR readout contrast for PL1 and PL5 defects.** The simulation curves are produced by fitting parameters in Eq. (2) of the main text that were fit to data extracted from various experiments (see text).

Conclusion.

Theoretical considerations imply that divacancy de-

fects embedded into quantum wells have significantly larger ODMR contrasts than those outside the quantum wells in 4H SiC. The energy gap between the singlet and triplet states is much smaller for divacancy defects in 4H SiC than that for diamond NV centre. Although this property results in a reduction in the ODMR contrast at room temperature for the divacancy defects in 4H-SiC, the room temperature off-resonant pulsed ODMR readout contrast values for PL5 and PL6 defects still approach about −26% on average with the applied setup and pulse protocol.

* These authors contributed equally to the work
† gali.adam@wigner.hu
‡ jsxu@ustc.edu.cn
§ cfli@ustc.edu.cn

References

[1] Li Q, Wang J-F, Yan F-F, Cheng Z-D, Liu Z-H, Zhou K, Guo L-P, Zhou X, Zhang W-P, Wang X-X, Huang W, Xu J-S, Li C-F and Guo G-C. Nanoscale depth control of implanted shallow silicon vacancies in silicon carbide. *Nanoscale* 2019; **11**: 20554-20561.

[2] Koehl WF, Buckley BB, Heremans FJ, Calusine G and Awschalom DD. Room temperature coherent control of defect spin qubits in silicon carbide. *Nature* 2011; **479**, 84-87.

[3] Falk AL, Buckley BB, Calusine G, Koehl WF, Dobrovitski VV, Politi A, Zorman CA, Feng PX-L and Awschalom DD. Polytype control of spin qubits in silicon carbide. *Nat Commun* 2013; **4**: 1819.

[4] Wang J, Zhou Y, Zhang X, Liu F, Li Y, Li K, Liu Z, Wang GZ and Gao WB. Efficient Generation of an Array of Single Silicon-Vacancy Defects in Silicon Carbide. *Phys Rev Appl* 2017; **7**: 064021.

[5] Wang J, Zhou Y, Wang Z, Rasmita A, Yang J, Li X, von Bardeleben HJ and Gao W. Bright room temperature single photon source at telecom range in cubic silicon carbide. *Nat Commun* 2018; **9**: 4106.

[6] Zhou Y, Wang Z, Rasmita A, Kim S, Berhane A, Bodrog Z, Adamo G, Gali A, Aharonovich I, and Gao W. Room temperature solid-state quantum emitters in the telecom range. *Sci Adv* 2018; **4**: eaar3580.

[7] Neumann P, Kolesov R, Jacques V, Beck J, Tisler J, Batalov A, Rogers L, Manson NB, Balasubramanian G, Jelezko F and Wrachtrup J. Excited-state spectroscopy of single NV defects in diamond using optically detected magnetic resonance.*New J Phys* 2009; **11**: 013017.

[8] Christle DJ, Klimov PV and de las Casas CF, Szász K, Ivády V, Jokubavicius V, Ul Hassan J, Syvddotajärvi M, Koehl WF, Ohshima T, Son NT, Janzén E, Gali Á and Awschalom DD. Isolated Spin Qubits in SiC with a High-Fidelity Infrared Spin-to-Photon Interface. *Phys Rev X* 2017; **7**: 021046.

[9] Dréau A, Lesik M, Rondin L, Spinicelli P, Arcizet O, Roch J-F and Jacques V. Avoiding power broadening in optically detected magnetic resonance of single NV defects for enhanced dc magnetic field sensitivity. *Phys Rev B* 2011; **84**: 195204.

[10] Yan F-F, Wang J-F, Li Q, Cheng Z-D, Cui J-M, Liu W-Z, Xu J-S, Li C-F and Guo G-C. Coherent Control of Defect Spins in Silicon Carbide above 550 K. *Phys Rev Appl* 2018; **10**: 044042.

[11] Ryan CA, Hodges JS and Cory DG. Robust Decoupling Techniques to Extend Quantum Coherence in Diamond. *Phys Rev Lett* 2010; **105**: 200402.

[12] Jarmola A, Acosta VM., Chemerisov S and Budker D. Temperature- and Magnetic-Field-Dependent Longitudinal Spin Relaxation in Nitrogen-Vacancy Ensembles in Diamond. *Phys Rev Lett* 2012; **108**: 197601.

[13] Christle DJ, Falk AL, Andrich P, Klimov PV, Ul Hassan J, Son NT, Janzén E, Ohshima T and Awschalom, DD. Isolated electron spins in silicon carbide with millisecond coherence times. *Nat Mater* 2015; **14**: 160-163.

[14] Anderson CP, Bourassa A, Miao KC, Wolfowicz G, Mintun PJ, Crook AL, Abe H, Ul Hassan J, Son NT, Ohshima T and Awschalom DD. Electrical and optical control of single spins integrated in scalable semiconductor devices. *Science* 2019; **366**: 1225-1230.

[15] Son NT, Carlsson P, ul Hassan J, Janzén E, Umeda T, Isoya J, Gali A, Bockstedte M, Morishita N and Ohshima T and Itoh H. Divacancy in 4H-SiC. *Phys Rev Lett* 2006; **96**: 055501.

[16] Wolfowicz G, Anderson CP, Yeats AL, Whiteley SJ, Niklas J, Poluektov OG, Heremans FJ and Awschalom DD. Optical charge state control of spin defects in 4H-SiC. *Nat Commun* 2017; **8**: 1876.

[17] Ivády V, Davidsson J, Delegan N, Falk AL, Klimov PV, Whiteley SJ. Hruszkewycz SO, Holt MV, Joseph Heremans F, Son NT, Awschalom DD, Abrikosov IA and Gali A. Stabilization of point-defect spin qubits by quantum wells. *Nat Commun* 2019; **10**: 5607.

[18] Gali Á. *Ab initio* theory of the nitrogen-vacancy center in diamond. *Nanophotonics* 2019; **8**: 1907-1943.

[19] Bockstedte M, Schütz F, Garratt T, Ivády V and Gali A. Ab initio description of highly correlated states in defects for realizing quantum bits. *npj Quantum Mater* 2018; **3**: 31.

[20] Thiering G and Gali A. Theory of the optical spin-polarization loop of the nitrogen-vacancy center in diamond. *Phys Rev B* 2018; **98**: 085207.

[21] Falk AL, Klimov PV, Buckley BB, Ivády V, Abrikosov IA, Calusine G, Koehl WF, Gali Á and Awschalom DD. Electrically and Mechanically Tunable Electron Spins in Silicon Carbide Color Centers. *Phys Rev Lett* 2014; **112**: 187601.
